# A new case of de novo 19p13.2p13.12 deletion in a girl with overgrowth and severe developmental delay

**DOI:** 10.1186/1755-8166-7-40

**Published:** 2014-06-05

**Authors:** Abdelhafid Natiq, Siham Chafai Elalaoui, Sevrine Miesch, Celine Bonnet, Philippe Jonveaux, Saaïd Amzazi, Abdelaziz Sefiani

**Affiliations:** 1 Département de Génétique Médicale, Institut National d’Hygiène, Rabat, Morocco; 2 Centre de génomique humaine, Faculté de médecine et de pharmacie, Université Mohammed V, Rabat, Morocco; 3 Faculté des Sciences de Rabat, Université Mohamed V Agdal, Rabat, Morocco; 4 Laboratoire de Génétique Médicale, Centre Hospitalier Universitaire, Nancy, France

**Keywords:** Overgrowth, Psychomotor delay, 19p13.2p13.12 deletion, Array comparative genomic hybridization

## Abstract

**Background:**

We report clinical and molecular cytogenetic characterization of a 2 year-old girl with 19p13.2p13.12 microdeletion and compare her clinical features with those of three other patients reported before.

**Result:**

Array comparative genomic hybridization (aCGH) revealed in the present patient a de novo microdeletion of 1.45 Mb within 19p13.2p13.12. The deletion includes seven OMIM genes: *MAN2B1, RNASEH2A, KLF1, GCDH, NFIX, CACNA1A and CC2D1A.*

**Discussion:**

The present case and three other patients with partially overlapping 19p13 microdeletion share the following features: psychomotor and language delay, intellectual disability, seizures, hypotonia, skeletal anomalies and facial dysmorphism. The smallest region of overlapping between all four reported patients is around 300 kb and spans only two genes: *NFIX* and *CACNA1A.* Their haploinsufficincy could be the base for the phenotype -genotype correlation.

## Background

Array comparative genomic hybridization (aCGH) has allowed for identification of the underlying molecular bases for numerous patients with multiple congenital anomalies. De novo microdeletion 19p13 detected by aCGH is rarely reported [1-4]. However, all yet known patients had intellectual disability and multiple congenital anomalies.

We report clinical and molecular cytogenetic characterization of a 2 year-old girl with a 19p13.2 to 19p13.12 microdeletion and compare her clinical features with those of three other patients [1,2,4]. The phenotype is mainly characterized by psychomotor and language delay, intellectual disability, seizures, hypotonia, skeletal anomalies and facial dysmorphism. The shortest region of overlap (SRO) extending for about 300 Kb between the four cases contains candidate genes responsible for their common phenotype (severe developmental delay, seizures, and skeletal anomalies). This microdeletion encompasses seven OMIM genes, among which two (*NFIX* and *CACNA1A*) could be the candidate genes for the genotype-phenotype relationship.

## Case presentation

### Case report

**Figure 1:**
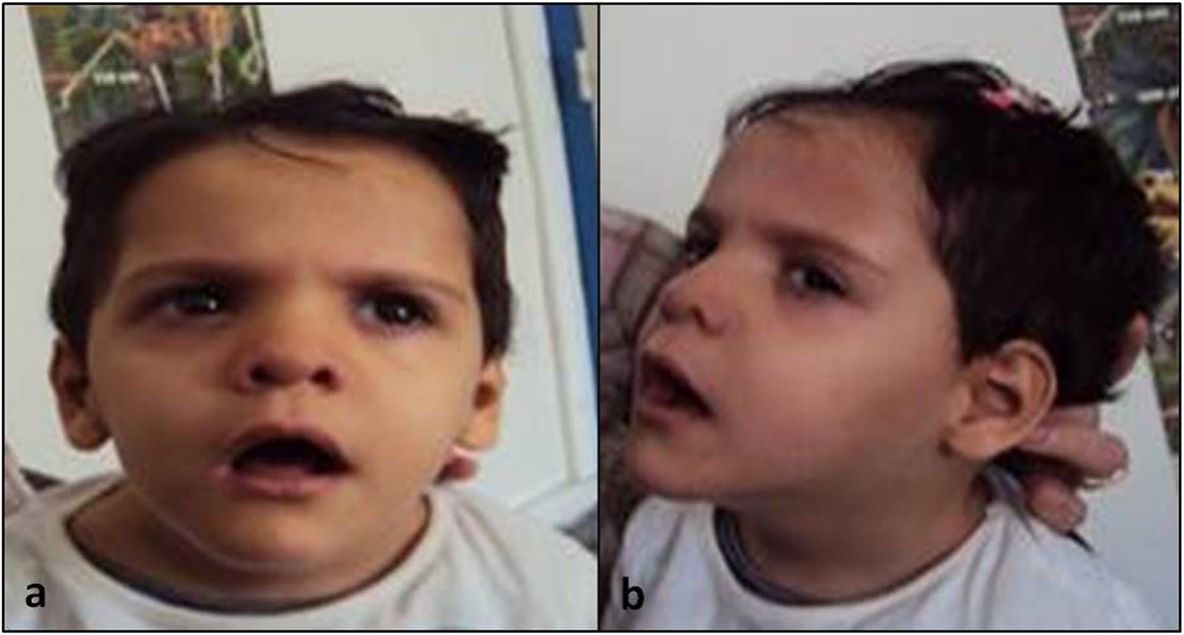
**Photographs of the patient at age of 2 years and 3 months, frontal (a) and lateral (b) view.** Note the tall forehead, anteverted nares, thin upper lip, anteverted ears and long philtrum.

The female patient was born at 36 weeks of gestational age to Moroccan consanguineous parents (first degree) with non-contributive familial history. She was referred to genetic consultation for suspicion of Angelman syndrome. She is the fifth liveborn to a 44-year-old mother and 55-year-old father. The mother was followed for chronic myeloid leukemia and was taking Imatinib during her pregnancy (400 mg/day). Still, pregnancy was without reported complications. Birth weight and length were 2,500 g and 42 cm respectively (both < 3rd percentile), head circumference was 31 cm (50th percentile). She had neonatal hypotonia and seizures since her birth. At clinical examination at fifteen months of age, her weight was 12 kg (>95th centile), and her head circumference 52 cm (>95th centile). She had severe axial hypotonia and was unable to hold her head or sit down in addition to absent speech. She also had facial dysmorphism including frontal bossing, down slanting palpebral fissures, microstomia, long philtrum, prognathia, anteverted nostrils, low-set ears, short neck (Figure 1) and also bilateral clinodactyly of the fifth finger.

At 2 years and 3 months her length was 97 cm (>95th centile), weight 15 kg (95th centile), and head circumference 52 cm (>95th centile). She was still hypotonic, unable to hold her head or to sit down, and with absent speech. The cerebral MRI showed bilateral frontal cortical atrophy. The EEG showed epileptiform focal abnormalities disorganized background rythm of slow (2-3 Hz) waves.

### Methods

Peripheral blood was collected from the patient and her parents. Informed consent was obtained from the patient’s parents prior to implementation of the genetic studies.

#### *Cytogenetics analysis*

Cytogenetics studies were performed on metaphase chromosome preparations obtained from phytohemagglutinin stimulated lymphocyte cultures according to standard procedures. Chromosome analysis was carried applying RHG banding at a 400-band level as previously reported and according to the International System for Human Cytogenetic: Nomenclature ISCN 2013 [5,6].

#### *DNA samples*

DNA was extracted from whole blood using the QIAmp DNA Kit (QIAGEN) according to the manufacturer’s instructions.

#### *Methyl PCR*

Methyl PCR was performed with Epitect Bisulfite Kit and EpiTect MSP kit according to the manufacturer’s instructions (QIAGEN).

#### *Array-based comparative genomic hybridization*

aCGH was carried out using 180 K-oligonucleotide array (Agilent, San Clara, CA) with an average resolution of about 25 kb, the procedure for DNA digestion and hybridization were performed according to the manufacturer’s instruction. For analysis of the result two databases were used respectively for the chromosomal localization: UCSC genome browser (http://genome.UCSC.edu) and for polymorphism control: database of genomic variants (http://dgv.tcag.ca/).

#### *Fluorescent in-situ hybridization (FISH) analysis*

FISH was performed on PHA-stimulated peripheral blood lymphocytes obtained from patient and both parents. BAC RP11-782D11 (located in 19p13.2p13.13) and CTD-2265021 (19q subtelomeric) probes obtained from CHORI (Oakland, CA) were used according standard protocols.

### Results

**Figure 2:**
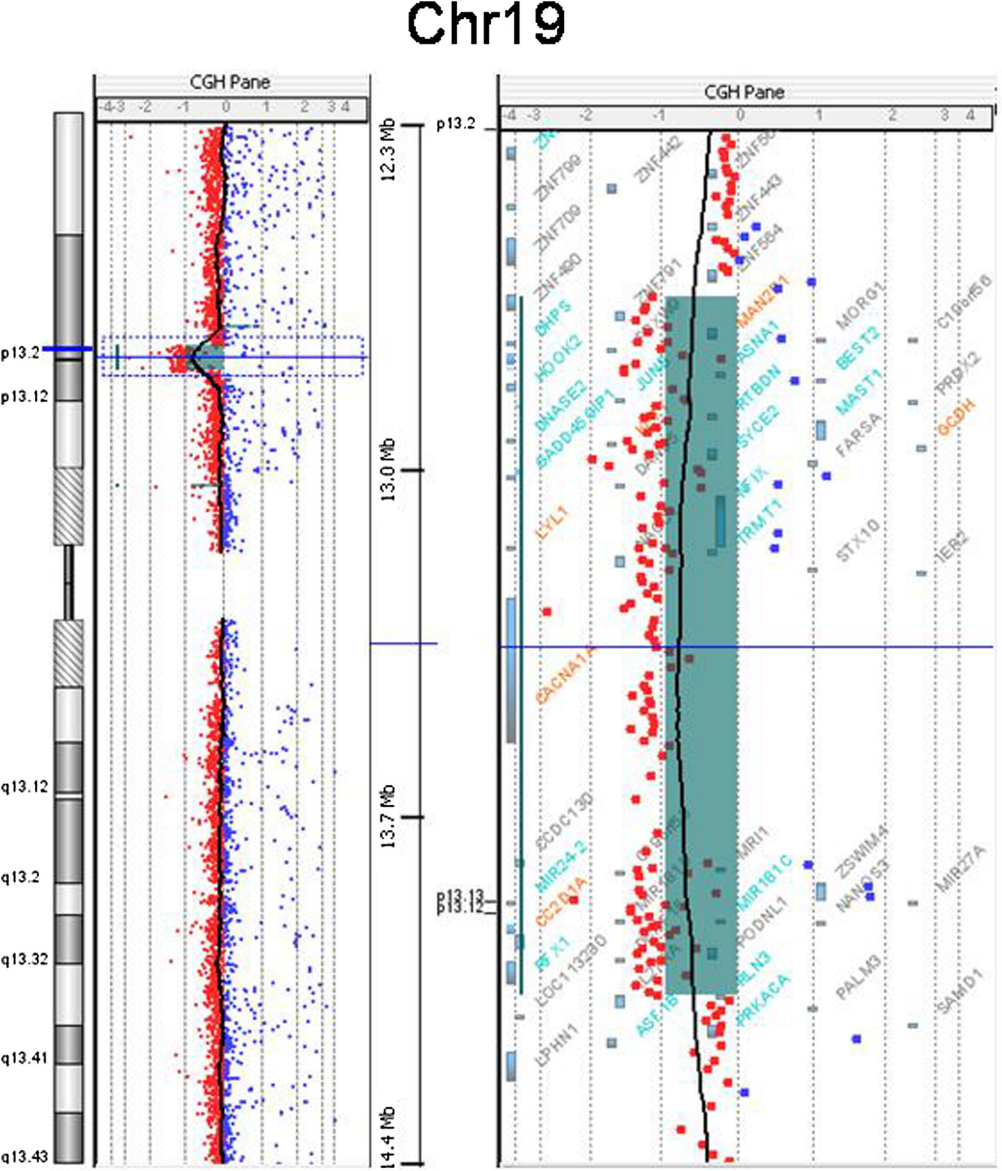
aCGH result shows the extend of the 19p13.2p13.12 deletion in the patient, with breakpoints at genomic positions 12,691,241-14,141,544 (GRCHh37/hg19).

**Figure 3:**
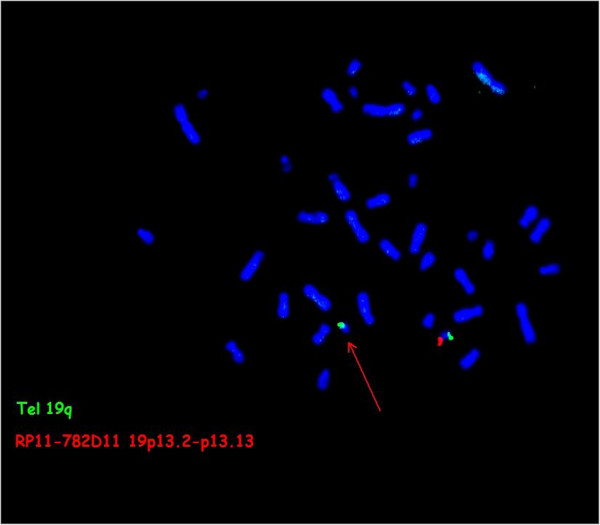
**FISH analysis of the 19p13.2-p13.12 deletion.** Spectrum-orange labeled BAC clone RP11-782D11 and spectrum-green labeled 19q subtelomeric (CTD-2265021) probe (Tel 19q) used for identification of chromosomes 19. Arrow indicate the absence of orange signal on chromosome del(19)(p13.2 ~ 13.12p13.2 ~ 13.12).

The karyotype was normal and presence of Angelman syndrome was excluded by methylation specific PCR (MSPCR) (data not shown). In the absence of any etiological diagnosis, array-CGH analysis identified a 1.45 Mb deletion at 19p13.2p13.12 in the patient (Figure 2): 46,XX.arr[hg19]19p13.2p13.12(12,691,241-14,141,544)x1, GRCHh37/hg19. The deleted region included 44 genes. Except for polymorphic regions, no copy number alterations were observed in other chromosomes. Because of the necessity to compare with other cases we reanalyzed with the hg 18 Build 36 version and the breakpoints were: 12,552,241-14,002,544. (NCBI36/hg18) The deletion was confirmed by FISH analysis (Figure 3), and is considered to be do novo since neither parent carries the deletion (data not shown). We report here, a 1.45 Mb microdeletion 19p13.2p13.12 in a child with overgrowth and multiple congenital anomalies. This deletion encompasses 44 genes from which only seven (*MAN2B1,RNASEH2A, KLF1, GCDH, NFIX, CACNA1A, and CC2D1A*) were OMIM genes and reportedly as involved in human diseases.

## Conclusion

Microdeletion 19p13 has been rarely reported in the literature [1-4]. This region has a high gene density and this is most likely the reason why deletions in this region are associated with a severe phenotype. Actually with the development of the chromosomal microarray analysis technology there has been a little increase in the number of reported cases with a microdeletion 19p13 [4]. The clinical features common to our patient and the cases reported before [1,2,4] are psychomotor and language delay, intellectual disability, seizures, hypotonia, skeletal anomalies and facial dysmorphism (Table 1).

**Table 1:** Summary of clinical features and cytogenetic characteristics of the reported patients with 19p13 deletion overlapping with our patient

**Patient**	**Present patient**	**Bonaglia et al. **[4] Patient 3	**Auvin et al. **[2]	**Lysy et al. **[1]	**Clinical score**
Gender	Female	Male	Male	Female	
Chrom.region	19p13.2-p13.12	19p13.13-p13.12	19p13.13	19p13.2-p13.13	
Deletion size Mb	1.45 Mb	1.5 Mb	664 Kb	3 Mb	
Position (hg18)	12.55-14.00	12.87-14.15	12.61-13.28	10.25-13.18	
At birth					
Weight	< 3rd	< 3rd	Normal	-2SD	
Length	< 3rd	< 3rd	Normal	-2SD	
OFC	50th	10-15th	Normal	-2SD	
At last evaluation					
Age	2^3/12^ years	7 years	2 years	3^8/12^ years	
Weight	>95 th	50th	+2SD	-2SD	
Length	95 th	75th	+3SD	-2SD	
OFC	95 th	50th	+2,5SD	-2SD	
Clinical features					
Hypotonia	Severe	+	+	+	4/4
Psychomotor delay	+	Moderate- severe	+	+	4/4
Language delay	+	+	+	+	4/4
Seizure/EEG anomalies	+	+	+	+	4/4
Hearing loss	-	Bilateral conductive	-	Bilateral threshold 60 dB	2/4
Skeletal	Advanced bone age	Scoliosis	Advanced bone age	Craniocynostosis with left spleno-orbital dysplasia	4/4
Extremities	Clinodactyly V	ClinodactylyV right hand, left I and II toes overlapping bracydactyly	-	-	2/4
Facial features					
Brachycephaly	+	+	-	-	2/4
Philtrum	long	long	-	-	2/4
Nose	Anteverted nares	Anteverted nares	Flat	-	3/4
Ocular anomalies	Strabismus	-	-	strabismus	2/4

**Figure 4:**
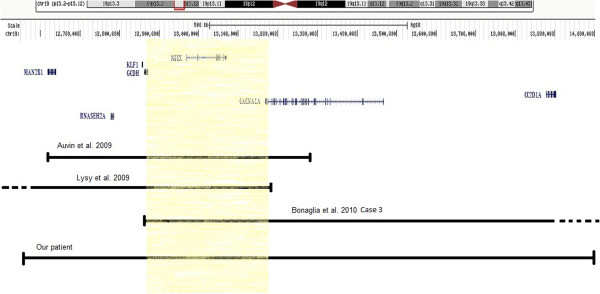
Schematic representation of 19p13.2-p13.12 region deleted in our patient and three other reported in the literature sharing about 300 kb indicated by color rectangle.

The four cases share approximately a 300-kb shortest region of overlap (SRO) including *NFIX1* and *CACNA1A* genes (Figure 4). This region had never been described as a copy number polymorphism (CNP) in the database of genomic variants (http://dgv.tcag.ca). *NFIX* [Nuclear Factor IX (CCAAT-binding transcription factor)] (OMIM 164005), a member of the nuclear factor I (Nfi) family of transcription factors, is highly expressed in the developing mouse brain and an essential gene for normal brain and skeletal development [5,7]. *NFIX* deficient mice show enlargement of the lateral and third brain ventricles and partial agenesis of the corpus callosum [7,8]. Our patient and the case of Auvin et al. [2] showed advanced bone age. Lysy et al. [1] reported craniosynostosis and kyphosis in their patients. The phenotype associated with *NFIX* haploinsufficiency may occur with variable penetrance because the third case reported by Bonaglia et al. [4] does not show any of these malformations. Other study demonstrates that *NFIX* deletion and its nonsense mutation (c.179T_C (p.Leu60Pro) and c.362G_C (p.Arg121Pro)) are associated with a novel clinically recognizable overgrowth syndrome like Marshall-Smith and Sotos like Syndrome [8,9]. The *CACNA1A* gene (calcium channel alpha 1A subunit) (OMIM 601011) is involved with the voltage–dependant Ca 2+ channels and has been reported in association with epilepsy and chronic neurological disorders, haploinsufficiency of this gene has been suggested to be responsible for epilepsy and infantile spasms [10,11]. Recently Marangi et al. report a deletion 19p12.13-p13.13 encompassing the first exon of the *CACNA1A* in a patient with EEG focal anomalies and suffering from febrile seizures [12]. In addition *CACNA1A* gene has been involved in the occurrence of developmental delay [13,14]. We suggest that *CACNA1A* gene deletion is likely responsible for the epileptic seizures in our patient.

In addition through this case report, we want to focus on the importance of a (aCGH) as a first-tier diagnostic step for patients with developmental disabilities and/or multiples congenital anomalies as reported by several scientific papers [15-18].

This report gives more information for the recently identified 19p13 deletion syndrome and clarifies the clinical implication of genes in the involved chromosomal region. Also, our paper may contribute to a better understanding of the genotype-phenotype correlation in cases with deletion in 19p13 and particularly in the involvement of *NFIX* and *CACNA1A* genes in overgrowth, epilepsies and developmental delay.

## Consent

Written informed consent was obtained from the patient’s parents for publication and accompanying images of this case report. A copy of the written consent is available for review by the Editor-in-Chief of this journal.

## Competing interest

The authors declare that they have no competing interests.

## Authors’ contribution

AN carried out the molecular genetic studies and drafted the manuscript. SCE helped to draft the manuscript, SM, CB and PJ participated in the molecular genetic studies and helped to draft the manuscript, SA participated in the design of the study and AS helped to draft the manuscript. All authors read and approved the final manuscript.
